# Clinical Efficacy of Transcutaneous Electrical Nerve Stimulation (TENS) in Pediatric Functional Constipation: Impact on Immunological Indicators and Gut Microbiota

**DOI:** 10.1155/jimr/8888730

**Published:** 2025-02-05

**Authors:** Piao Guo, Xue Ning Zhang, Xin Yu Jin, Wen Juan Xia, Li Zhou, Wei Song Sheng, Dan Rong Zhu

**Affiliations:** ^1^Department of Pediatrics, The Second Affiliated Hospital, Nanjing Medical University, Nanjing 210000, China; ^2^Department of Epidemiology Research, Jiangsu Health Development Research Center, Nanjing 210036, China; ^3^Department of Spleen and Stomach, Yangzhou Hospital of Traditional Chinese Medicine Affiliated to Nanjing University of Chinese Medicine, Yangzhou 225002, China; ^4^Department of Paediatrics, Yili Friendship Hospital, Nanjing Medical University, Ili 835000, China

**Keywords:** children, functional constipation, gut microbiota, immunological indicators, transcutaneous electrical nerve stimulation (TENS)

## Abstract

**Objective:** This study was conducted to evaluate the effectiveness of transcutaneous electrical nerve stimulation (TENS) in treating pediatric functional constipation (FC) and to explore its mechanisms by analyzing changes in serum neurotransmitters and gut microbiota.

**Materials and Methods:** This was a prospective cohort study conducted on 60 children aged 4–14 years diagnosed with FC. Participants were divided into two groups, namely, one receiving TENS therapy three times a week for 4 weeks and the control group receiving lactulose. Pretreatment and posttreatment evaluations included serum neurotransmitters, immunological indicators, and gut microbiota composition.

**Results:** The TENS group demonstrated significant improvements in defecation frequency and constipation symptoms compared with the lactulose group (*p* < 0.001). Posttreatment, remarkable increases were detected in serum motilin and vasoactive intestinal peptide (VIP) levels, along with a significant decrease in interleukin-12 levels (*p* < 0.05), indicating anti-inflammatory effects. Gut microbiota analysis revealed significant shifts in microbial composition, with an increase in the abundance of Bacteroidetes and a decrease in the abundance of Firmicutes/Bacteroidetes ratio, suggesting improved gut health and metabolic function.

**Conclusion:** TENS effectively improves symptoms of pediatric FC and induces beneficial changes in immunological indicators and gut microbiota. These results suggest potential anti-inflammatory and microbiota-modulating effects. However, due to the limited sample size, further studies are needed to confirm these findings and explore the long-term benefits of TENS therapy.

**Trial Registration:** Clinical Trial Registry identifier: ChiCTR2200059549

## 1. Introduction

Functional constipation (FC) in children is a prevalent gastrointestinal disorder that manifests through symptoms such as infrequent bowel movements, hard stools, and abdominal discomfort. Despite its commonality, the pathophysiological underpinnings, influenced by diet, behavior, and physical activity levels, remain incompletely elucidated. Recent studies highlight a worrisome increase in the global incidence of FC among children, currently estimated at 14.4%, indicating a 4.9% increase since 2018 [1, 2].

The repercussions of FC are far-reaching. On a physiological level, it can precipitate conditions such as mucosal damage from hard stools and anal fissures and, in severe instances, require surgical intervention [3]. Furthermore, FC may compromise immune function, increase the vulnerability to respiratory infections, and potentially impede growth. Psychologically, the distress and social withdrawal arising from FC can severely impact a child's mental health and developmental trajectory, with constipation-related behavioral problems affecting more than one-third of this population, thereby exerting considerable pressure on families and healthcare infrastructures [4, 5].

Despite the gravity of the implications of FC, gaps persist in our understanding of its pathogenesis and the optimization of management strategies. Current clinical protocols, inclusive of the Rome IV criteria, advocate for interventions spanning lifestyle and dietary adjustments, cognitive therapies, and pharmacological treatments. However, conventional approaches, especially laxatives, are ineffective for ~25% of pediatric patients, with a remarkable incidence of symptom recurrence posttherapy.

Against this background, transcutaneous electrical nerve stimulation (TENS) presents a viable alternative. Leveraging mild electrical impulses to activate nerve fibers, TENS has demonstrated potential in ameliorating conditions such as pain and fecal incontinence [6–9]. Early investigations indicate its applicability in pediatric FC, although its underlying mechanisms—possibly mirroring those of acupuncture through neural and humoral modulation—await detailed investigation [10–12]. Studies on adults have suggested the role of TENS in augmenting vagal nerve activity and facilitating intestinal peristalsis [13], and its capacity to adjust plasma compound levels might synchronize gastric myoelectric activity with the vagus nerve, thereby improving gastrointestinal motility [14]. The intricate relationship between elevated inflammatory markers and the gut microbiome further enriches this process, revealing that children with FC often exhibit signs of a subclinical inflammatory state. This observation suggests that changes in the composition of specific microbiota may be associated with increased inflammation, which could contribute to the pathophysiology of FC [15]. Hence, no firm conclusions regarding the efficacy and safety of TENS in children with chronic constipation can be drawn. Further randomized controlled trials evaluating TENS for the management of pediatric constipation are necessary. Future trials should include clear documentation of methodologies, especially measures to evaluate the effectiveness of blinding, and incorporate patient-important outcomes such as the number of patients with improved complete spontaneous bowel movement (CSBM) and improved clinical symptoms and quality of life [16].

This study was conducted to bridge these knowledge gaps by investigating the clinical effectiveness of TENS in the management of pediatric FC and clarifying its mechanisms of action. By analyzing the serum levels of vasoactive intestinal peptide (VIP), MTL, interleukin (IL)-6, and IL-12 and using 16S rRNA and macrogene high-throughput sequencing before and after TENS treatment, we intend to clarify the impact of TENS on the gut microbial ecosystem, flora density, and metabolomics. Our objective was to ascertain whether TENS can mitigate FC symptoms by modulating the intestinal microbiota and metabolic pathways, thereby restoring gut homeostasis.

## 2. Materials and Methods

### 2.1. Study Design and Patient Enrollment

This prospective cohort study included 76 pediatric patients from the Friendship Hospital of Ili Prefecture, Xinjiang, and the Second Affiliated Hospital of Nanjing Medical University. Recruitment was conducted between June 1, 2021, and December 31, 2022. Ethical approval was obtained from the Ethics Committee of the Second Affiliated Hospital of Nanjing Medical University (Approval No. YY2021-0041), with informed written consent obtained from the guardians of all participants.

#### 2.1.1. Grouping Methods

A randomized block group design was used to allocate the 76 children to the lactulose and TENS groups for the intervention on a trial basis, using the duration of illness as a matching factor, and 4 children with FC adjacent to the duration of illness as a block group. This was performed as follows: (1) The 76 children with FC were numbered in the order of disease duration. (2) Every four children with similar disease duration were assigned to a unit group and numbered. (3) The design was accomplished using the SPSS software. The random seed was set (when the random seed is fixed, a fixed random number is generated). The “random number generator” was converted, and the fixed value was entered. (4) Random numbers were generated. The “random number” was entered, and the function “Rv. Uniform” was used. (5) Finally, the lactulose and TENS groups each contained 38 patients. However, due to participant dropout during the study, the final analysis included 60 children (30 in each group).

#### 2.1.2. Case Inclusion or Exclusion Criteria

The inclusion criteria were as follows: (1) children aged 4–14 years, (2) diagnosed with FC according to the Rome IV criteria, (3) exclusion of constipation induced by food allergies (e.g., milk and eggs), (4) ability to comply with the trial's follow-up requirements, (5) no use of laxatives or other medications affecting bowel function within the past month, and (6) BMI between 18.5 and 24 kg/m^2^. The exclusion criteria were as follows: (1) history of upper respiratory tract infections (e.g., colds and fever) within the past month, (2) history of gastrointestinal infections (e.g., abdominal pain, diarrhea, and vomiting) within the past month, (3) history of urinary tract infections (e.g., dysuria and hematuria) within the past month, (4) use of medications that may affect the gut microbiota in the past month (e.g., probiotics, prebiotics, gastric acid inhibitors, and antibiotics), (5) presence of underlying chronic diseases, and (6) inability to adhere to the study follow-up schedule.

### 2.2. Treatment Protocol

Children in the TENS group were treated using a KD-2B transcutaneous electrical nerve stimulator (Beijing Yaoyang Kangda Medical Instrument Co., Ltd.) three times a week for at least 30 min each time. The sites were selected at the umbilical level below the costal margin of the anterior abdominal wall (2 inches near the navel) and between the ninth thoracic vertebra and second lumbar vertebra beside the spine of the back. The four sites were simultaneously stimulated in a symmetrical pattern, and the stimulation parameters were set at a frequency of 5 Hz and an intensity of 10 mA. Children in the control group were orally administered lactulose (0.7 g/kg/day, Beijing Hanmei).

### 2.3. Sample Collection

Venous blood (3 mL) was collected from fasting participants and centrifuged (3000 rpm, 10 min), and the resulting serum was stored at −80°C. Fecal samples were collected in disposable bowls, transferred to collection tubes without air exposure, and stored at −80°C. Additionally, stool samples from five children within the same TENS group, who also experienced symptom relief, were subjected to 16S rRNA and metagenomic high-throughput sequencing.

### 2.4. Enzyme-Linked Immunosorbent Assay (ELISA)

Serum concentrations of the target biomarkers were measured using a commercial ELISA kit according to the manufacturer's instructions. Briefly, standards and samples were added to precoated wells and incubated at 37°C. After washing, the enzyme substrates were added, followed by incubation and detection at 450 nm. The assay's sensitivity ranged from 0.5 to 6 ng/mL. All measurements were performed in duplicate to ensure accuracy. Detailed protocols are available in the standard ELISA procedures [17].

### 2.5. 16S rRNA Sequencing and Analysis

Ten fecal samples collected from five children were preserved on dry ice and analyzed by Shanghai Majorbio Bio-Pharm Technology Co., Ltd. Whole genomic DNA was extracted from the fecal samples, and its concentration and purity were evaluated by 1% agarose gel electrophoresis. The V3–V4 variable regions of the 16S rRNA gene were amplified using the primers 388F and 806R. After PCR, the products were verified and size-selected by 2% agarose gel electrophoresis. Gel recovery of the PCR products was performed using the AxyPrepDNA Gel Recovery Kit (AXYGEN Inc.), followed by elution in Tris-HCl and further verification by 2% agarose gel electrophoresis. Libraries were prepared using the NEXTFLEX Rapid DNA-Seq Kit and sequenced on the Illumina MiSeq PE300 platform. Raw sequences were processed for quality control using Fastp, merged using the Flash software, and cleaned of host genome-associated reads using BWA. OTU clustering was performed against the Silva 16S rRNA database (v138) with a 97% similarity threshold using the Uparse software. Taxonomic classification of representative sequences from each OTU was performed using the RDP Classifier with a confidence threshold of 0.7, facilitating the analyses of species composition and abundance.

### 2.6. Macrogenomic Analysis

Genomic DNA was isolated from 10 fecal samples collected from five children using the E.Z.N.A. Soil DNA Kit (Omega Bio-tek, USA), according to the manufacturer's protocol. The concentration and purity of DNA were quantified using a TBS-380 fluorometer and a NanoDrop 2000 spectrophotometer, respectively. DNA integrity was confirmed by 1% agarose gel electrophoresis, ensuring an average fragment size of ~400 bp after shearing using Covaris M220, suitable for downstream library construction. Paired-end libraries were prepared using the NEXTFLEX Rapid DNA-Seq Kit. High-throughput sequencing was performed on an Illumina NovaSeq/Hiseq Xten platform using NovaSeq/HiSeq X Reagent Kits for optimal coverage and depth. Raw sequences, stored in fastq format, were subjected to stringent quality control via Fastp, filtering out reads less than 50 bp, with average quality scores <20, or containing N bases. The BWA software was used to eliminate reads aligning with host DNA. High-quality reads supported the assembly of macrogenomic data using MEGAHIT, based on succinct de Bruijn graph principles, selecting contigs ≥300 bp as final assembly outcomes. Open reading frames within contigs were predicted using Metagene. CD-HIT was used to cluster predicted genes at 95% sequence identity, and SOAPaligner was used to quantify gene abundance against the representative sequences. Nonredundant gene sets were annotated against the NR database using DIAMOND for species identification and abundance estimation. The KEGG database provided functional annotation, using diamond pairs for KEGG Orthology (KO) group assignments, facilitating pathway enrichment and gene function analysis. Community diversity indices (Chao, Shannon, Ace, and Simpson) and principal coordinates analysis (PCoA) were used to evaluate microbial diversity and community structure variations before and after TENS treatment, supported by PERMANOVA and LEfSe for statistically significant differences and biomarker discovery. Metabolic pathway variations between the groups were identified using Wilcoxon rank-sum tests, indicating relevant microbial genes within the KEGG pathways.

### 2.7. Fecal Untargeted Metabolomics Analysis and Data Processing

Fecal samples (100 µL) were mixed with 400 µL acetonitrile:methanol (1:1), sonicated at 5°C and 40 kHz for 30 min, and centrifuged at 13,000 rpm and 4°C after a 30-min rest at −20°C. The resulting supernatant was dried, reconstituted in 100 µL acetonitrile:water (1:1), and then prepared for chromatographic analysis. Using a UHPLC-Q Exactive system with an HSS T3 column, we used a dual-phase system, consisting of 95% water with 0.1% formic acid and 47.5% acetonitrile with 5% water, also containing 0.1% formic acid. The operational parameters included a flow rate of 0.40 mL/min and a column temperature of 40°C. Mass spectrometry was performed in dual ion modes across a 70–1050 m/z range, optimizing the detection through the tailored ion spray voltage, sheath and auxiliary gas pressures, and variable collision energy. QC samples, created by pooling metabolites from all samples, were analyzed to validate the consistency of the analytical process. The Progenesis QI software was used to process the LC-MS raw data, facilitating peak identification, alignment, and normalization. Identification of metabolites was achieved by matching the spectra with HMDB, Metlin databases, and an in-house library. The final data matrix was refined through meticulous filtering, normalization, and QC validation steps. Normalized data were subjected to differential analysis using fold change and statistical tests, with significant metabolites identified via OPLS-DA (VIP level > 2, *p* < 0.005). The KEGG pathway analysis revealed metabolic alterations and network interactions. Spearman's correlation was used to examine the relationships between the differential species, metabolites, and metabolic pathways, which revealed critical biological insights.

### 2.8. Statistical Analysis

Statistical analyses were performed using SPSS version 26. For normally distributed data, the efficacy of treatments was evaluated using a general linear model, with results expressed as mean ± standard deviation (SD) and 95% confidence intervals (CIs). For nonparametric data, the Mann–Whitney *U* test was used, with results presented as median (interquartile range [IQR]). Categorical variables were reported as frequencies and percentages (*N* [%]). A *p*-value of <0.05 was considered statistically significant.

## 3. Results

### 3.1. Clinical Outcomes of TENS

In this cohort of 60 pediatric patients, the demographic and baseline characteristics (age, gender, and duration of constipation) were comparable across the TENS and lactulose groups, indicating well-matched groups at the study outset (Table 1).

Our longitudinal analysis revealed a significant improvement in stool frequency in the TENS group compared with that in the lactulose group across the 4-week period, with the TENS group showing a more marked increase from the baseline to week 4 (Table 2). By the second week, patients in the TENS group showed a significant increase in defecation frequency by 55% (95% CI: 51.42%–58.58%, *p* < 0.01) compared to baseline, along with a reduction in painful bowel movements by 40% (95% CI: 36.42%–43.58%, *p* < 0.01).

The TENS treatment also resulted in a significant reduction in the symptoms associated with FC. By week 4, there was a remarkable decrease in painful bowel movements and large-diameter feces in the TENS group, highlighting its effectiveness in symptom management (Table 3).

**Figure 1:**
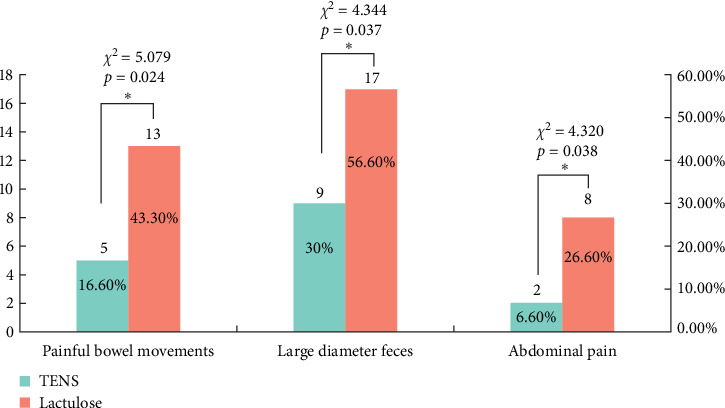
Symptom comparison at the end of week 4 (*p* < 0.05 marked with *⁣*^*∗*^).

The TENS group showed a significant reduction in symptoms, with a remarkable decrease in painful bowel movements and large-diameter feces by the end of the study (Figure 1).

According to the Rome IV criteria, effective treatment is defined as more than two bowel movements per week, passing formed soft stools, and the disappearance of concomitant symptoms such as painful bowel movements and abdominal pain. Although the median number of bowel movements was significantly higher in the control group (4 [2, 6]) than in the TENS group (3 [2, 5]) in most of the children in the first week, all 27 (90%) children in the TENS group responded to treatment in week 1 compared to 24 (80%) children in the control group. Moreover, 5 (13.3%) children in the control group had unformed stools, whereas none in the TENS group had unformed stools. During treatment, 10 (33.3%) children in the lactulose group did not respond at 0.7 g/kg or responded for a short time and required an increase in the dose. At the end of the study, 25 (83.3%) children in the TENS group had a PAC-QOL score (≥9) compared to 20 (66.6%) in the lactulose group. A follow-up at week 4 after the end of treatment (week 8 of the trial) revealed that 20 (66.6%) children remained in the TENS group compared to 10 (33.3%) in the lactulose group, and at the week 8 follow-up, children in the TENS group had a bowel movement. The frequency of defecation was 5 (3, 7) in the TENS group and 5 (4, 6) in the control group at the week 8 follow-up, with a statistical difference of *p*=0.025.

### 3.2. Biochemical Markers

**Figure 2:**
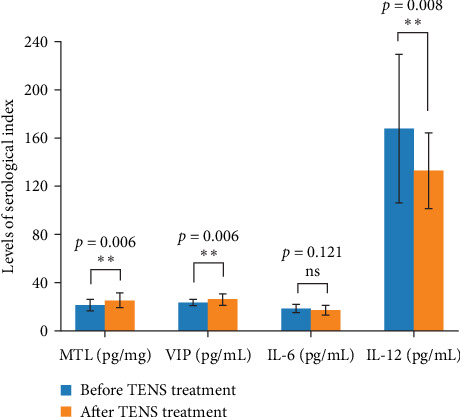
Levels of the serological index before and after TENS treatment (*p* < 0.01 marked with *⁣*^*∗∗*^, *p* ≥ 0.05 marked with “ns”).

Posttreatment, there were significant increases in serum MTL and VIP levels, along with a remarkable reduction in IL-12 levels, indicating the anti-inflammatory effects of TENS treatment (Figure 2). After TENS treatment, serum VIP levels increased by 38% (*p*=0.02), Motilin levels increased by 45% (*p*=0.01), and IL-12 levels decreased by 30% (*p*=0.03) compared to baseline.

### 3.3. Microbiota Diversity

**Figure 3:**
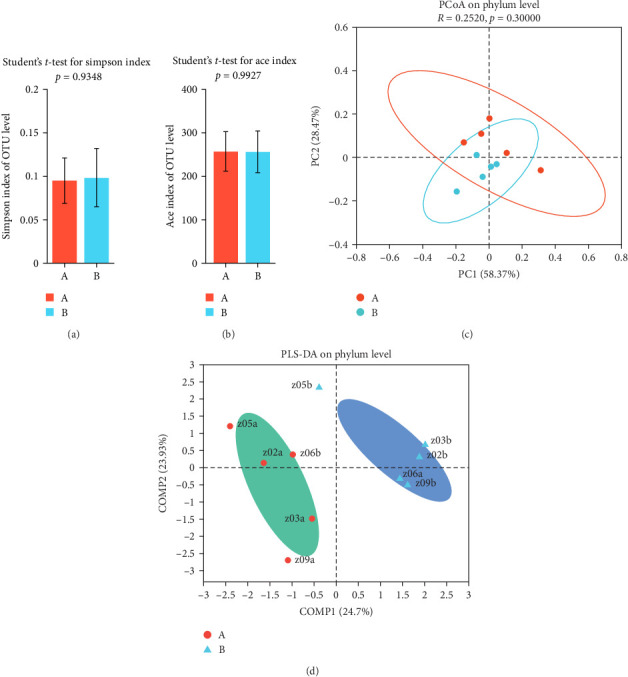
Effect of transcutaneous electrical nerve stimulation (TENS) on the intestinal flora of children with functional constipation (FC) (A: samples of children with FC before TENS treatment; B: samples of children with FC after TENS treatment). (a) Simpson index; (b) Ace index; (c) PCoA; and (d) PLS-DA.

No significant change was detected in the diversity of gut microbiota after TENS treatment, as indicated by the Chao, Shannon, and Ace indices. However, specific microbial taxa differentiation was observed, emphasizing the nuanced impact of TENS on the composition of gut microbiota (Figure 3).

### 3.4. Microbial Abundance

**Figure 4:**
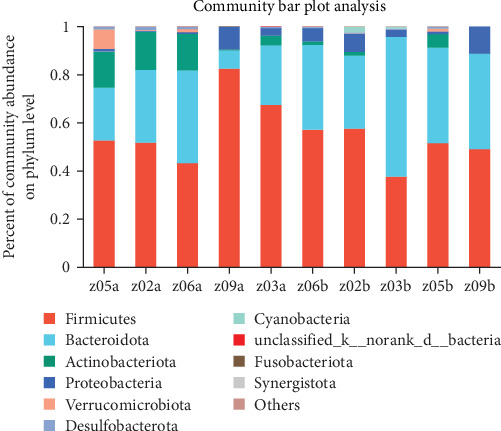
Phylum-level composition of the dominant phylum in groups A and B (A: samples of children with functional constipation [FC] before transcutaneous electrical nerve stimulation [TENS] treatment; B: samples of children with FC after TENS treatment).

**Figure 5:**
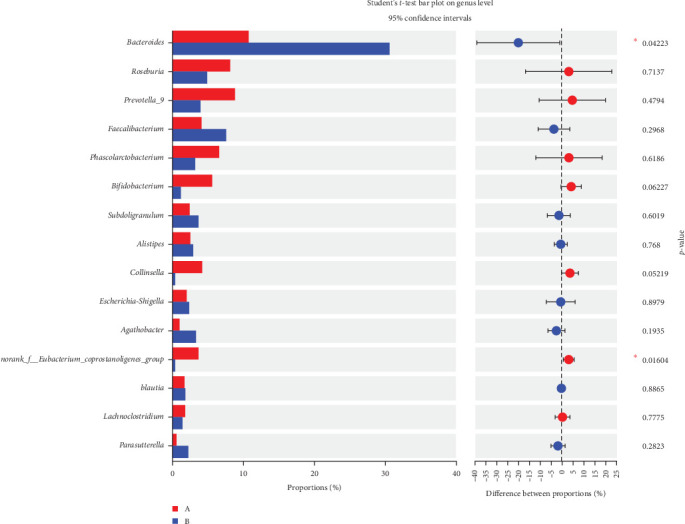
Community structure of the intestinal flora before and after TENS treatment (*p* < 0.05 marked with *⁣*^*∗*^).

**Figure 6:**
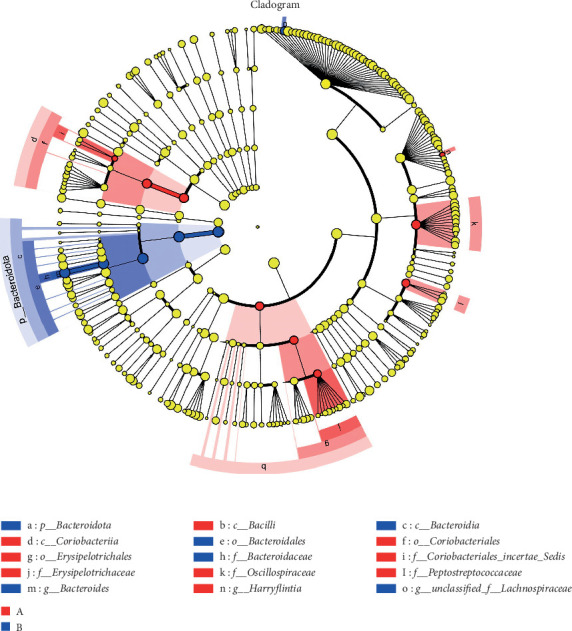
LEfSe multilevel species hierarchy tree diagram.

Significant shifts were detected in microbial abundance at the phylum level, with a 20% increase in the abundance of Bacteroidetes and a 15% decrease in the Firmicutes/Bacteroidetes ratio in the TENS group after treatment (*p*=0.04), indicating a shift toward a healthier gut microbiome (Figures 4–6, Table 4)

### 3.5. Impact of TENS Treatment on KEGG Functional Pathways in Intestinal Microbiota

**Figure 7:**
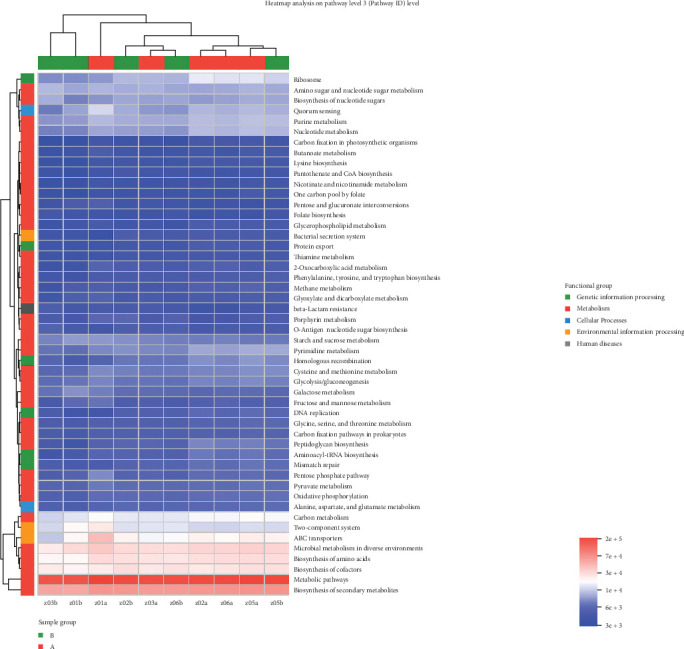
KEGG functional composition heat map.

**Figure 8:**
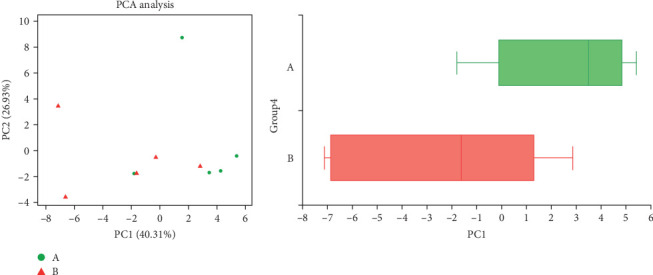
PCA between the groups.

**Figure 9:**
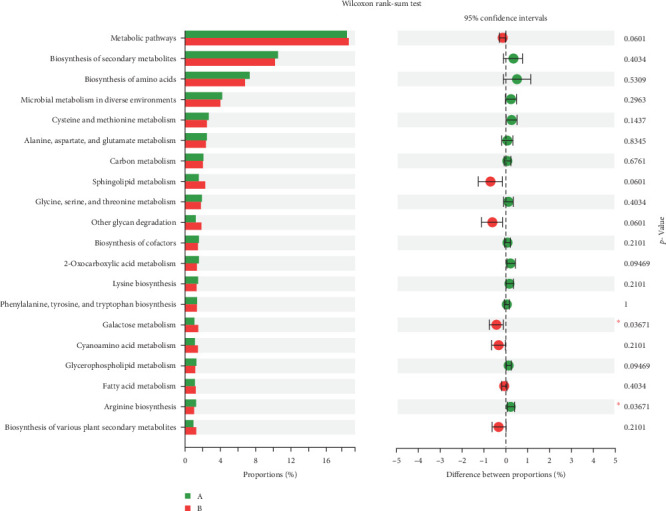
Structure of the KEGG functional pathway (*p* < 0.05 marked with *⁣*^*∗*^).

**Figure 10:**
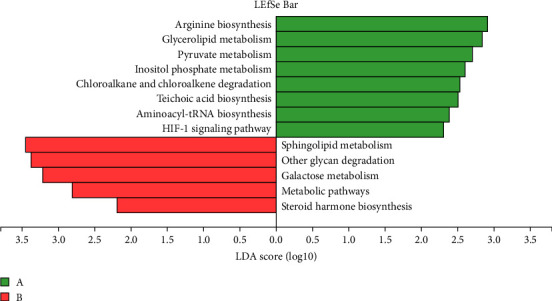
Discriminant analysis of the functional pathway significance between groups.

The comparative analysis of KEGG functional pathways revealed distinct patterns of microbial gene expression before and after TENS treatment. The shift in microbial functionality is visually summarized in the heat map (Figure 7) and principal component analysis (Figure 8), denoting significant alterations in specific pathways. In particular, posttreatment samples demonstrated an increased abundance of galactose metabolism and a decreased presence of the arginine biosynthesis pathway (Figure 9), indicating the targeted impact of TENS. The differential abundance of these pathways was statistically significant (*p* < 0.05). Furthermore, the linear discriminant analysis indicated the most influential pathways contributing to the observed variations (Figure 10). Pretreatment enrichment included pathways such as arginine biosynthesis and glycerolipid metabolism, whereas posttreatment observations highlighted sphingolipid metabolism and steroid hormone biosynthesis, indicating a marked metabolic shift induced by TENS therapy.

### 3.6. Analysis of Species and Their Functional Contributions

**Figure 11:**
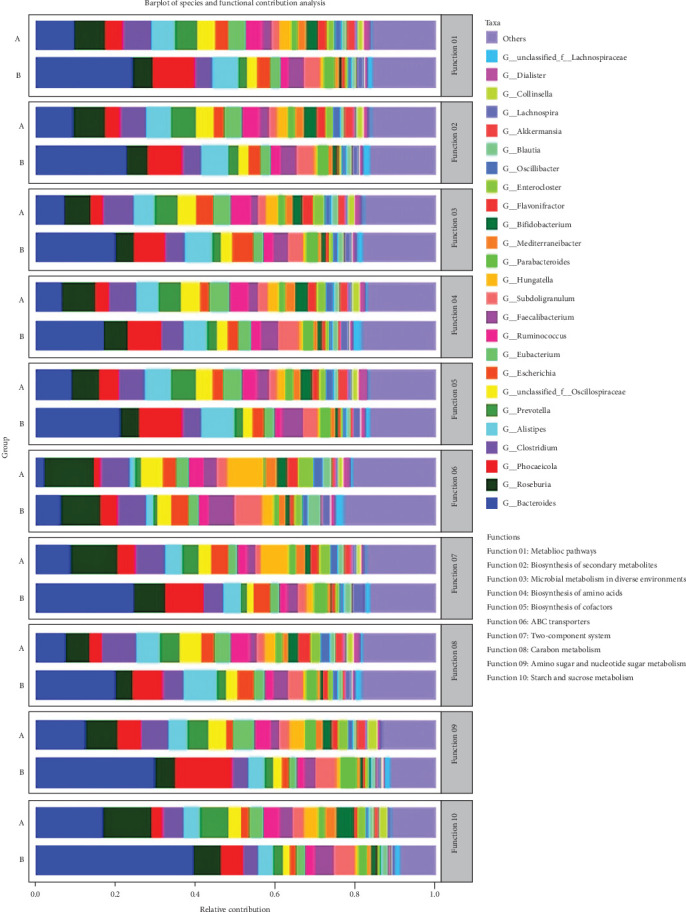
Analysis of gut microbial genus level and functional contribution.

Our analysis revealed a significant enhancement in the role of *Bacteroides* in the posttreatment group, particularly in contributing to metabolic pathways, as shown in Figure 11. Specifically, after TENS therapy, the increased abundance of *Bacteroides*, *Faecalibacterium*, and *Lachnospira* was found to be closely associated with changes in specific KEGG pathways at level 3. These genera showed a higher contribution to differential pathway activations, indicating that the shifts in KEGG pathway activities were directly linked to the observed changes in the microbial composition after treatment. This emphasizes the substantial impact of TENS therapy on the functional capacity of the gut microbiota.

### 3.7. Fecal Metabolomic Profiling

**Figure 12:**
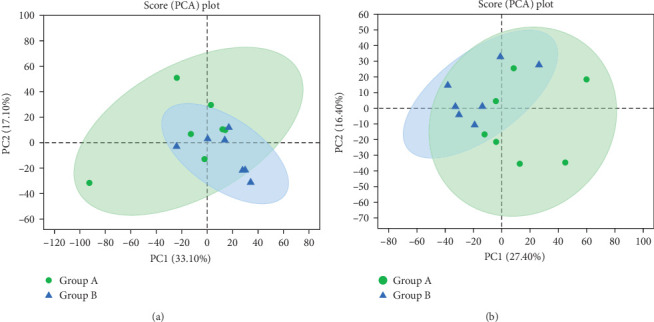
(A) PCA in the positive ion mode and (B) PCA in the negative ion mode.

**Figure 13:**
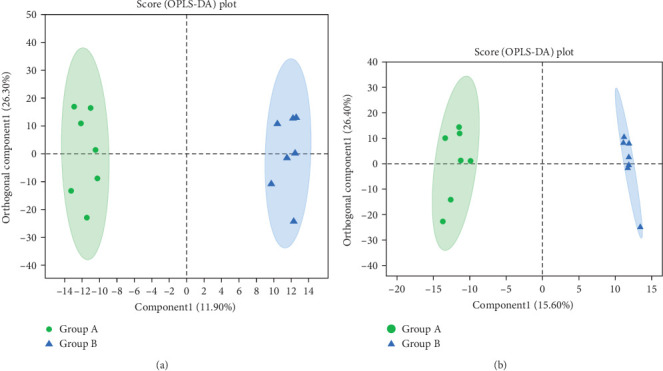
(A) OPLS-DA score graph in the positive ion mode and (B) OPLS-DA score graph in the negative ion mode.

**Figure 14:**
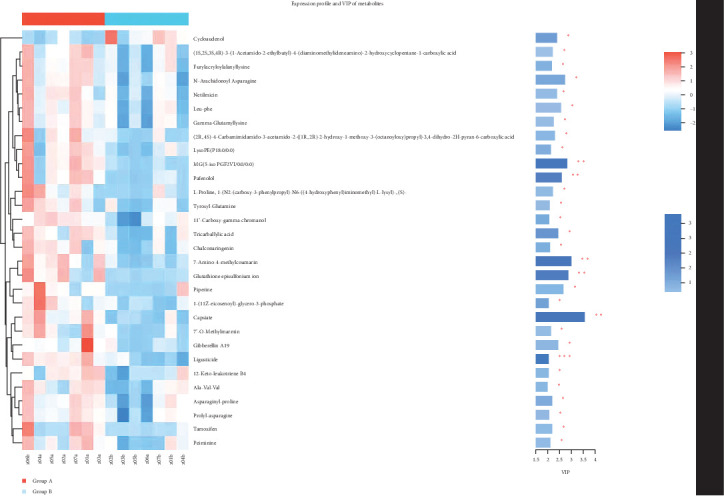
Heat map of the differential metabolites (*p* < 0.05 marked with *⁣*^*∗*^, *p* < 0.01 marked with *⁣*^*∗∗*^).

The results of PCA revealed obvious distinctions between the two sample groups, as illustrated in Figure 12. Moreover, the results of OPLS-DA analysis indicated a significant differentiation between samples from group A (before TENS treatment) and group B (after TENS treatment), as depicted in Figure 13. Through OPLS-DA analysis screening, we identified metabolites with VIP level > 2 (paired *t*-test, *p* < 0.005) as differential, as shown in Table 5. The heat map analysis further demonstrated substantial differences in the distribution of metabolites between the two groups of fecal samples, as depicted in Figure 14.

### 3.8. Pathway Enrichment Analysis of Differential Metabolites

**Figure 15:**
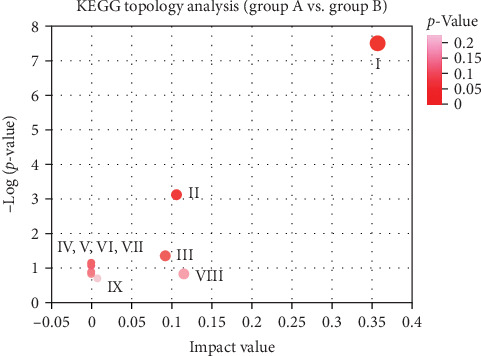
KEGG topology analysis of the differential metabolites.

The differential metabolites identified in this study were subjected to separate KEGG pathway enrichment and KEGG topology analyses, which revealed the following eight metabolic pathways that exhibited significant involvement: cutin, suberine, and wax biosynthesis (I), caprolactam degradation (II), prodigiosin biosynthesis (III), α-linolenic acid metabolism (IV), unsaturated fatty acid biosynthesis (V), cytochrome P450 metabolism of exogenous substances (VI), fatty acid biosynthesis (VII), and flavonoid biosynthesis (VIII). These results are depicted in Figure 15.

### 3.9. Association Analysis Between Differential Metabolites and Bacterial Genera

**Figure 16:**
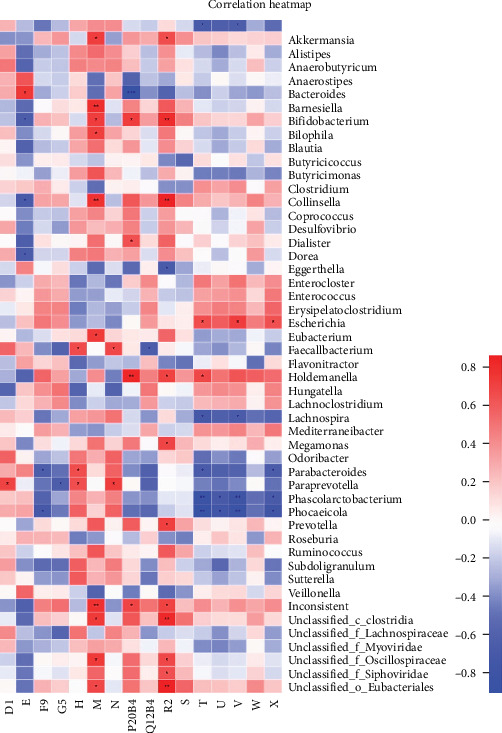
Heat map of correlation between differential metabolites and differential genera (*p* < 0.05 marked with *⁣*^*∗*^, *p* < 0.01 marked with *⁣*^*∗∗*^, and *p* < 0.001 marked with *⁣*^*∗∗∗*^).

**Figure 17:**
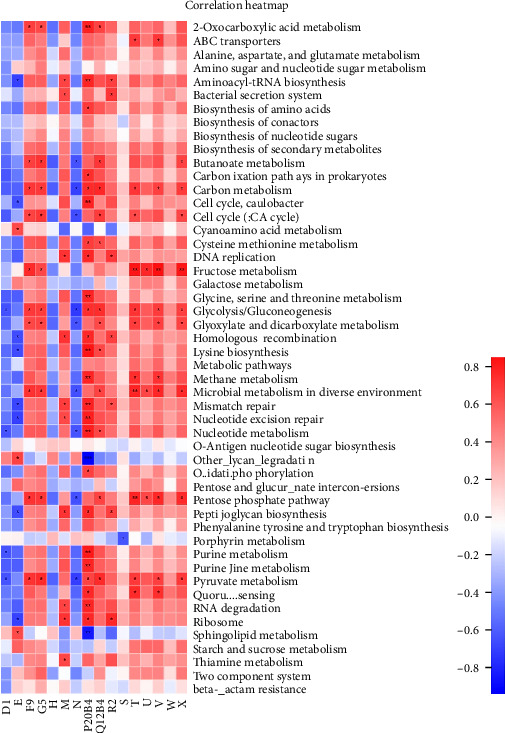
Heat map of correlation between differential metabolites and differential functional pathways annotated with KEGG (*p* < 0.05 marked with *⁣*^*∗*^, *p* < 0.01 marked with *⁣*^*∗∗*^, and *p* < 0.001 marked with *⁣*^*∗∗∗*^).

To explore the interaction between microbial species and metabolites, we conducted a heatmap analysis to determine the Spearman correlation coefficients, effectively highlighting the relationships following TENS treatment.  Figure 16 illustrates these relationships at the genus level, showing significant correlations between specific bacterial genera and differential metabolites. Similarly, Figure 17 shows the relationships between the differential metabolites and the KEGG metabolic pathways, highlighting the biological significance of the identified metabolites such as 1-hexanol (D1), fluoride ionic acid (E), and 9-oxononanoic acid (F9). These findings provide a comprehensive overview of metabolic interactions within the gut microbiome influenced by TENS therapy, further exemplified by specific metabolites such as arachidonic acid (H) and adipic acid (M).

## 4. Discussion

This study provides novel insights into the dual benefits of TENS for symptom relief and altering gut microbiota composition in pediatric patients with FC, suggesting a substantive enhancement over conventional lactulose therapies. Compared to lactulose, TENS demonstrated superior efficacy, particularly in enhancing stool frequency and consistency while effectively reducing symptoms of painful defecation and abdominal pain. The TENS group exhibited a 55% increase in defecation frequency (95% CI: 51.42%–58.58%, *p*  < 0.01) and a 40% reduction in painful defecation (95% CI: 36.42%–43.58%, *p*  < 0.01) from the second week onward, underscoring its potential as a primary or adjunct therapy for pediatric FC management [6–10, 18–20]. These observations align with prior findings, including studies on electrical stimulation techniques' effectiveness in gastrointestinal motility disorders, reinforcing TENS's viability in FC management [18–24].

Our findings revealed significant biochemical shifts post-TENS treatment, including increases in serum VIP by 38% (*p*=0.02) and MTL by 45% (*p*=0.01), alongside a 30% reduction in IL-12 levels (*p*=0.03). VIP enhances gastrointestinal motility by relaxing smooth muscles, promoting secretion, and increasing blood flow, which facilitates stool passage [19, 22]. Elevated MTL levels stimulate peristalsis, which is crucial for alleviating constipation [24]. The reduction in IL-12 levels indicates an anti-inflammatory effect of TENS, potentially alleviating subclinical inflammation in children with FC and improving mucosal health.

Gut microbiota analysis revealed significant compositional shifts following TENS treatment, including a 20% increase in the relative abundance of Bacteroidetes and a 15% decrease in the Firmicutes/Bacteroidetes ratio (*p*=0.04), indicating a shift toward a healthier gut microbial profile [25–28]. This shift could enhance intestinal motility through the production of short-chain fatty acids (SCFAs), which are known to stimulate intestinal peristalsis and improve gut barrier function [27, 28]. However, no significant changes in overall microbial diversity (Shannon index, *p* > 0.05) were observed, possibly due to the relatively short intervention duration of 4 weeks, which may be insufficient for significant diversity changes to manifest [15, 18]. This suggests that TENS may selectively influence specific bacterial taxa rather than broadly altering microbial diversity.

Interestingly, TENS treatment may also influence the abundance of Proteobacteria, which has been implicated in gastrointestinal motility. Wu et al. [29] discovered that lipopolysaccharide (LPS) from Proteobacteria could enhance gastrointestinal motility by activating toll-like receptor 4 (TLR4), thereby increasing spontaneous contractions and acetylcholine responses in the ileum and colon. Furthermore, He et al. [30] demonstrated through Mendelian randomization that alterations in gut microbiota composition could causally influence constipation, suggesting that specific genera play a direct role in the condition's pathophysiology. Our analysis posits that TENS could restore gastrointestinal motility by increasing the relative abundance of Proteobacteria, although the 4-week duration might not have allowed for complete recovery and stabilization of gut flora. Recovery of intestinal flora is a complex process potentially influenced by individual differences, lifestyle, and diet, necessitating longer-term follow-up to comprehensively assess TENS's effects [25, 29, 30].

In addition to the compositional changes, metabolomic analysis revealed significant alterations in metabolic pathways post-TENS treatment. We observed increased galactose metabolism and decreased arginine biosynthesis, suggesting that TENS influences microbial metabolic functions relevant to gastrointestinal motility and inflammation [27, 28]. Modulation of these pathways may contribute to the therapeutic effects of TENS by enhancing beneficial metabolic activities while reducing proinflammatory processes. Furthermore, changes in metabolite levels, including increased 1-hexanol and decreased fatty acids, support the impact of TENS on lipid metabolism, which may influence both motility and immune responses [14, 29].

Despite these promising results, the study has several limitations. Due to limited funding, the small sample size for microbiota analysis (only five samples for 16S rRNA sequencing) may restrict the generalizability of the findings. Future studies should include larger sample sizes and comprehensive sequencing of both treatment and control groups to validate these observations [16, 21, 25]. Additionally, the 4-week treatment duration may not fully capture the long-term effects of TENS on gut microbiota and FC symptoms. Previous research suggests that microbiota stabilization requires extended periods, highlighting the need for longer follow-up studies to assess the durability of TENS-induced changes [22, 23]. Moreover, while significant biochemical and microbial compositional changes were observed, the lack of significant shifts in overall microbial diversity warrants further investigation. It is possible that TENS primarily exerts targeted effects on specific taxa, as suggested by the significant changes in Bacteroidetes and Firmicutes abundance. Future research should explore the functional implications of these selective changes, particularly in relation to SCFA production and their role in gut motility [15, 24].

## 5. Conclusion

In summary, TENS therapy demonstrates significant potential as a nonpharmacological treatment for pediatric FC, with improvements in stool frequency, biochemical markers, and gut microbiota composition. However, further studies with larger sample sizes and longer follow-up periods are necessary to validate these findings and to better understand the mechanisms underlying TENS-induced changes in gut function. These efforts will help establish TENS as a reliable and effective treatment modality for pediatric FC, potentially reducing the need for pharmacological interventions and improving the quality of life for affected children.

## Figures and Tables

**Table 1:** Baseline demographic profile and constipation parameters.

Parameters	TENS (*n* = 30) (*n*, %)	Lactulose (*n* = 30) (*n*, %)	*p*-Value
Age (mean ± SD)	7.32 ± 1.964	7.12 ± 2.28	0.722
Gender			
Male	13 (43.3%)	16 (53.3%)	0.438
Female	17 (56.7%)	14 (46.7%)	—
Duration of constipation	18.00 (12, 26)	18.00 (11, 28)	0.755
Defecation ≤2 times/week	13 (43.3%)	14 (46.7%)	0.795
Painful bowel movements	18 (60.0%)	16 (53.3%)	0.602
Large diameter feces	17 (56.7%)	14 (46.7%)	0.438

*Note:* The duration of constipation is expressed as the median (min, max). Gender, ≤2 bowel movements per week, painful bowel movements, and large diameter stools, expressed as *n* (%).

Abbreviations: SD, standard deviation; TENS, transcutaneous electrical nerve stimulation.

**Table 2:** Longitudinal ANOVA analysis of stool frequency.

Treatment modality	Baseline week (times)	Week 1 (times)	Week 2 (times)	Week 3 (times)	Week 4 (times)	*p*-Value
TENS (*n* = 30, mean ± SD, 95% CI)	2.6 ± 1.037 (2.21, 2.99)	3.33 ± 0.758 (3.05, 3.62)	5.50 ± 1.042 (5.19, 6.01)	6.23 ± 1.455 (5.65, 6.75)	6.30 ± 1.043 (5.80, 6.80)	= 0.001
Lactulose (*n* = 30, mean ± SD, 95% CI)	2.47 ± 0.973 (2.10, 2.83)	4.07 ± 1.081 (3.66, 4.47)	5.00 ± 1.083 (4.60, 5.40)	5.23 ± 0.858 (4.91, 5.55)	5.37 ± 1.098 (4.96, 5.78)	

Abbreviations: SD, standard deviation; TENS, transcutaneous electrical nerve stimulation.

**Table 3:** Mann–Whitney *U* test analysis of weekly bowel movement frequency (median [minimum, maximum]).

Treatment modality	Baseline week (times)	Week 1 (times)	Week 2 (times)	Week 3 (times)	Week 4 (times)
TENS (*n* = 30)	3 (1, 4)	3 (2, 5)	6 (3, 7)	6 (3, 8)	6 (4, 8)
Lactulose (*n* = 30)	3 (1, 4)	4 (2, 6)	5 (3, 7)	5 (3, 7)	5.5 (4, 7)
*p*-Value	0.633	0.007	0.035	0.005	0.007

Abbreviation: TENS, transcutaneous electrical nerve stimulation.

**Table 4:** The dominant bacterial genera in the intestine of groups A (before TENS treatment) and B (after TENS treatment).

Group	Species name	Mean	LDA value	*p*-Value
A	*p__Actinobacteriota.c__Coriobacteriia.o__Coriobacteriales.f__Coriobacteriales_Incertae_Sedis*	2.171794317	3.845007663	0.034266056

A	*p__Actinobacteriota.c__Coriobacteriia.o__Coriobacteriales*	4.651343454	4.261816462	0.047201768

A	*p__Firmicutes.c__Bacilli.o__Erysipelotrichales.f__Erysipelotrichaceae*	3.739462182	3.327470403	0.047201768

A	*p__Firmicutes.c__Clostridia.o__Oscillospirales.f__Oscillospiraceae*	4.797153545	4.188513632	0.047201768

A	*p__Actinobacteriota.c__Coriobacteriia*	4.651343454	4.261816462	0.047201768

A	*p__Firmicutes.c__Clostridia.o__Oscillospirales.f__Ruminococcaceae.g__Harryflintia*	1.908552883	4.199420122	0.016827409

A	*p__Firmicutes.c__Bacilli.o__Erysipelotrichales*	3.918570003	3.457979008	0.009023439

A	*p__Firmicutes.c__Clostridia.o__Peptostreptococcales-Tissierellales.f__Peptostreptococcaceae*	3.778761643	3.397510808	0.036145142

A	*p__Firmicutes.c__Bacilli*	4.088726564	3.588863166	0.028280123

B	*p__Firmicutes.c__Clostridia.o__Lachnospirales.f__Lachnospiraceae.g__unclassified_f__Lachnospiraceae*	3.875085696	3.461641686	0.047201768

B	*p__Bacteroidota.c__Bacteroidia*	5.608780518	4.905528686	0.028280123

B	*p__Bacteroidota.c__Bacteroidia.o__Bacteroidales.f__Bacteroidaceae.g__Bacteroides*	5.486073405	4.935714369	0.028280123

B	*p__Bacteroidota.c__Bacteroidia.o__Bacteroidales.f__Bacteroidaceae*	5.486073405	4.935714369	0.028280123

B	*p__Bacteroidota*	5.608780518	4.905528686	0.028280123

B	*p__Bacteroidota.c__Bacteroidia.o__Bacteroidales*	5.608766083	4.905492749	0.028280123

Abbreviation: TENS, transcutaneous electrical nerve stimulation.

**Table 5:** Identification of 16 relevant differential metabolites and trends based on LC-MS.

Metabolites	Library ID	M/Z	VIP predicted OPLS-DA	*p*-Value	FC (group A/group B)
1-Hexanol	HMDB0012971	246.2425904	1.1937	0.02812	0.9684
Fluoride ionic acid	HMDB0034295	315.2526576	1.0309	0.008662	0.9747
9-Oxononanoic acid	HMDB0094711; LMFA01060160	367.2087602	1.3747	0.03372	1.0637
5-Methoxyindoleacetic acid salt	HMDB0004096; PW_C002060	188.0704957	1.1705	0.008707	1.0374
Peanut acid	HMDB0002212; LMFA01010020	330.3362706	1.2878	0.04852	0.952
Adipic acid	LMFA01170048; HMDB0000448	145.049845	1.5287	0.01112	1.076
Dodecanoic acid	pw_c000497; hmdb0303347; hmdb0002262; lmfa01010012; hmdb0000638; hmdb0303348	218.2113216	1.4217	0.01883	0.9566
20 Carboxylated leukotriene B4	HMDB0006059; PW_C002644; LMFA03020016	365.196803	1.8358	0.03512	1.1408
12-Keto-leukotriene B4	PW_C002098; HMDB0004234	379.2125008	2.0172	0.03409	1.1637
2-Propylglutaric acid	HMDB0060684; PW_C040548	173.0813391	1.1128	0.03722	1.0553
Glutathione cyclic sulfide ion	HMDB0060479	357.0995984	2.8618	0.004326	1.2955
Furoacryloyl lysine	HMDB0247373	336.1565548	2.1706	0.02981	1.1907
Asparaginyl proline	HMDB0028739	262.1395348	2.1697	0.02537	1.1636
Threonine lysine	HMDB0029064	233.1494315	1.9642	0.04923	1.1656
Tyrosine glutamine	HMDB0029103	292.132106	2.065	0.04847	1.188
Prolinyl asparagine	HMDB0029012	262.1395277	2.0639	0.04819	1.1617

Abbreviation: FC, functional constipation.

## Data Availability

The original contributions presented in the study are included in the article. Further inquiries can be directed to the corresponding authors or the first author. Data will be made available on request.
